# Abstinence-Dependent Effects of Long-Access Cocaine Self-Administration on Nucleus Accumbens Astrocytes Are Observed in Male, But Not Female, Rats

**DOI:** 10.1523/ENEURO.0310-22.2022

**Published:** 2022-09-07

**Authors:** Ronald Kim, Anze Testen, Eden V. Harder, Natalie E. Brown, Emily A. Witt, Tania J. Bellinger, Janay P. Franklin, Kathryn J. Reissner

**Affiliations:** 1 Department of Psychology and Neuroscience, University of North Carolina at Chapel Hill, Chapel Hill, NC 27599; 2 Neuroscience Curriculum, University of North Carolina at Chapel Hill, Chapel Hill, NC 27599; 3 Pharmacology Graduate Program, University of North Carolina at Chapel Hill, Chapel Hill, NC 27599

**Keywords:** astrocyte, cocaine, morphology, nucleus accumbens, sex differences, synaptic colocalization

## Abstract

Accumulating evidence indicates significant consequences for astrocytes associated with drug abuse. For example, reductions in structural features and synaptic colocalization of male rat nucleus accumbens (NAc) astrocytes are observed following short-access (ShA; 2 h/d) self-administration and extinction from cocaine, methamphetamine, and heroin. However, it is unknown whether these observations extend to other rodent models of drug abuse, how enduring these effects may be, and whether similar effects are observed in female rats. Here, we assess the effects of long-access (LgA; 6 h/d) cocaine self-administration and abstinence on NAc astrocytes separately in male and female rats, employing a commonly used behavioral approach to investigate the incubation of cocaine craving. NAc astrocytes from male rats exhibit extensive (∼40%) reductions in surface area, volume, and postsynaptic colocalization 45 d but not 24 h after the last self-administration session. In contrast, no effect of self-administration and abstinence was observed in astrocytes from female rats. Moreover, no effect of LgA self-administration and abstinence was observed on NAc GLT-1 expression in female rats, an effect that has been well described in males. These results indicate striking and sexually dimorphic effects of abstinence subsequent to LgA self-administration on astrocytes. Taken together, these results indicate a pivotal role of prolonged abstinence in the effects of cocaine self-administration on NAc astrocytes, and extend a growing body of evidence regarding sex differences in the cellular consequences of drug self-administration in the brain.

## Significance Statement

Previous studies have reported changes in astrocytes following short-access (ShA; 2 h/d) cocaine self-administration and extinction. However, it is unknown whether these changes occur following other rodent models of drug intake. Accordingly, we examined the morphometric properties and synaptic colocalization of nucleus accumbens (NAc) astrocytes in male rats following long-access (LgA; 6 h/d) cocaine self-administration and prolonged abstinence (45 d). NAc astrocytes demonstrated a profound decrease (∼40%) in surface area, volume, and synaptic colocalization. Importantly, these changes are greater following LgA and abstinence, versus ShA and extinction. Furthermore, these changes are not observed 24 h following LgA cocaine self-administration and not observed in female rats. These results indicate abstinence and sex-dependent changes in NAc astrocytes following prolonged abstinence from LgA cocaine self-administration.

## Introduction

Astrocytes are characterized by their unique structural features, which include complex, fine peripheral processes. These processes can contact thousands of synapses, thereby enabling astrocytes to bidirectionally communicate with neurons and modulate neuronal activity (Perea et al., 2009; Durkee and Araque, 2019). This bidirectional communication between astrocytes and synapses subserves important functions within the central nervous system including neurotransmitter uptake, ion homeostasis, synapse development, and synaptic transmission and plasticity (Allen and Barres, 2009; Sofroniew and Vinters, 2010; Nedergaard and Verkhratsky, 2012; Clarke and Barres, 2013). Disruptions in these interactions and functions carry significant implications for neuronal function and behavior (Acosta et al., 2017; Booth et al., 2017) and represent contributing mechanisms of neuropsychiatric diseases (Scofield and Kalivas, 2014; Peng et al., 2015; Blanco-Suárez et al., 2017; R. Kim et al., 2018a).

Disruption in neuron-astrocyte communication is also observed following exposure to drugs of abuse. In particular, chronic downregulation of astroglial regulators of glutamate homeostasis, glutamate transporter GLT-1 and catalytic subunit of the cystine glutamate exchanger xCT, are observed in the nucleus accumbens (NAc) following cocaine self-administration and either extinction or abstinence in rats (Knackstedt et al., 2010; Fischer-Smith et al., 2012; Fischer et al., 2013; R. Kim et al., 2018b). Importantly, pharmacological restoration of these proteins inhibits reinstatement of cocaine seeking (Reissner et al., 2014, 2015; LaCrosse et al., 2017; Sepulveda-Orengo et al., 2018).

Downregulation of structural features of NAc astrocytes is also observed following cocaine self-administration and extinction training, including surface area, volume, and colocalization with both presynaptic and postsynaptic markers (Scofield et al., 2016; Testen et al., 2018). Furthermore, these changes in astrocytes are specific to the NAc and are not observed 24 h after the last self-administration session (Testen et al., 2018). Similar decreases in the synaptic colocalization of NAc astrocytes are observed following methamphetamine (Siemsen et al., 2019) and heroin (Kruyer et al., 2019) self-administration and extinction. These studies collectively suggest that drug self-administration leads to reduced capability of NAc astrocytes to modulate neuronal synapses, which may in turn lead to increased relapse vulnerability. In support of this hypothesis, stimulating NAc astrocytes with Gq-DREADDs decreases both cocaine reinstatement (Scofield et al., 2015), and ethanol self-administration (Bull et al., 2014). Furthermore, stimulation of astrocyte DREADD signaling in the reward circuitry alters ethanol drinking (Erickson et al., 2021; Nwachukwu et al., 2021). Specifically, agonism of prefrontal cortical astrocyte hM3D Gq receptors has been reported to increase drinking in ethanol-naive mice (Erickson et al., 2021), but reduce drinking in a binge paradigm when ligand is administered systemically or directly to the BLA (Nwachukwu et al., 2021).

The current study was designed to investigate astrocyte adaptations in male and female rats in a self-administration protocol associated with the incubation of cocaine craving. In comparison to the short-access (ShA)/extinction model of rodent cocaine self-administration, the incubation of cocaine craving model has been hypothesized to provide a translationally-relevant model for drug craving in human addicts (Pickens et al., 2011; Li et al., 2015). This model typically (but not necessarily) employs long-access (LgA) cocaine self-administration (6 h/d) followed by prolonged abstinence (30–45 d). The hallmark feature of incubation is an increase in cue-induced drug seeking following a period of home-cage abstinence (Tran-Nguyen et al., 1998; Grimm et al., 2001; Lu et al., 2004b; Ferrario et al., 2005). Cue-induced drug seeking is significantly higher following LgA (vs ShA), and significantly increased after abstinence (vs extinction; Lu et al., 2004b; Ferrario et al., 2005). Interestingly, this increase in drug seeking is further augmented with longer abstinence periods, with the highest amount of drug seeking behavior exhibited at ∼45 d of abstinence, before beginning to decline after 60 d (Lu et al., 2004b; Pickens et al., 2011). Importantly, the incubation of drug craving has also been observed in human substance use disorders (Bedi et al., 2011; Wang et al., 2013; Parvaz et al., 2016), underscoring the translational relevance of this model.

Structural features and synaptic colocalization of astrocytes were assessed using a validated approach which employs a membrane-associated lymphocyte protein kinase green fluorescent protein (Lck-GFP) virus under the control of the astrocyte-specific GfaABC1D promoter (Scofield et al., 2016; Testen et al., 2018). Lck-GFP allows for visualization and characterization of the fine peripheral processes of astrocytes which are not observed using glial fibrillary acidic protein (GFAP) immunostaining or cytosolic astrocytic GFP (Shigetomi et al., 2010, 2013). Synaptic colocalization of astrocytes was also assessed using immunohistochemistry for the postsynaptic marker PSD-95, as described previously using the ShA/extinction model (Testen et al., 2018).

In addition to assessment of LgA self-administration and prolonged abstinence on NAc astrocytes, this study investigated effects of LgA cocaine self-administration and abstinence on NAc GLT-1 and structural features of astrocytes in female rats. A reduction in GLT-1 has been reported in the NAc following ShA/extinction in female rats, as seen in male rats (Bechard et al., 2018); however, it is unknown whether these findings extend to the incubation of cocaine craving model. Moreover, no studies to date have investigated the effects of cocaine self-administration on structural features of female astrocytes.

## Materials and Methods

### Animals and surgical procedures

Male (225–250 g) and female (200–225 g) Sprague Dawley rats were purchased from Envigo, individually housed in temperature and humidity controlled standard Plexiglas cages on a reverse light-dark cycle (7 A.M. off, 7 P.M. on). All rats were allowed to acclimate to the animal facility for one week, with food and water available ad-libitum. All rats were then placed on a food restricted diet of ∼20 g of chow per day. Food restriction lasted throughout all surgical, postoperative, and food-training procedures. Rats were then returned to an ad-libitum diet during self-administration which lasted throughout the duration of the study. For all female rats, estrous cycle stage was determined on the day of euthanasia using previously defined methods (Marcondes et al., 2002). All procedures were approved by the University of North Carolina at Chapel Hill Institutional Animal Care and Use Committee and followed the National Institutes of Health *Guide for the Care and Use of Laboratory Animals*.

For surgical procedures, rats were anesthetized with ketamine (100 mg/kg) and xylazine (7 mg/kg), and a silastic catheter was implanted into the right jugular vein as previously described (R. Kim et al., 2018b; Sepulveda-Orengo et al., 2018). Meloxicam (4 mg/kg, i.p.) was provided on the day of surgery, as well as 24 and 48 h after surgery. Catheters were flushed daily with gentamicin (5 mg/ml, 0.1 ml) and heparinized saline (100 U/ml, 0.1 ml) throughout all postoperative and self-administration procedures. Immediately following jugular vein catheterization in the same surgery, rats were microinjected with Lck-GFP under the control of the GfaABC1D promoter, packaged into the AAV5 serotype (6.1 × 1012 virus particles/ml) by the University of North Carolina (UNC) Viral Vector Core as previously described (Shigetomi et al., 2013; Scofield et al., 2016; Testen et al., 2018, 2019). Bilateral microinjections targeted the NAc (6° angle, AP +1.5, ML +2.6, DV −7.2) and virus was microinjected (0.1 μl/min, 1 μl per hemisphere) using 26-gauge microinjection cannulas (Plastics One). Microinjectors were left in place for 15 min to allow for virus diffusion and then slowly removed over 1–2 min. Before the start of self-administration procedures, patency of the catheters was examined by administering a subthreshold dose of propofol (10 mg/ml, 0.05 ml).

### Self-administration procedures

All self-administration procedures took place in standard sound-attenuated operant conditioning chambers (Med Associates). Before the start of self-administration, all animals received one food-training session, where responding on the active lever resulted in the delivery of one 45-mg food pellet (Bio Serv). Food training sessions lasted a minimum of 6 h and criteria for food training was set at >100 responses on the active lever. Rats then received 10 d of saline (0.9% NaCl) or cocaine (5 mg/ml, 0.75 mg/kg/infusion) self-administration on an FR1 schedule for 6 h/d for 10 consecutive days (typically 42–45 μl per infusion for a male rat, 35–38 μl per infusion for a female rat). Responding on the active lever resulted in the delivery of saline or cocaine (0.045 ml/infusion for a 300-g rat, over 2.18 s), accompanied by a tone (70 dB, 2.5 kHz) and illumination of a stimulus light above the active lever for 5 s. A 20-s timeout period occurred after every infusion, where active lever responding during this time resulted in no programmed responses. Infusions were capped at 80 for the first 2 d, and subsequently at 200 for the remainder of the self-administration phase. Responding on the inactive lever at any time resulted in no programmed responses. Following 10 d of self-administration, animals remained in the home cage for 45 d and were handled twice per week.

### Immunohistochemistry

For astrocyte imaging experiments, rats were euthanized 24 h following the last day of LgA self-administration or 24 h following the last day of abstinence. All rats were deeply anesthetized with sodium pentobarbital and transcardially perfused with 1× phosphate buffer (PB), followed by 4% paraformaldehyde (PFA; in PB). Brains were extracted, postfixed in 4% PFA for ∼4 h and then stored in 30% sucrose. Tissue sections (100 μm) from the NAc were collected using a cryostat (Leica Biosystems) and stored in 50% glycerol/PBS until staining.

For immunohistochemistry staining, free floating NAc sections were first washed (3 × 5 min) in 1× PBS containing 2% Triton X-100 (PBST; Thermo Fisher Scientific). Sections were then blocked in 5% normal goat serum (NGS; Sigma-Aldrich) in PBST for 1 h at room temperature. Blocking solution was then replaced with primary antibodies [mouse anti-PSD-95 (Thermo Fisher Scientific) and rabbit anti-GFAP (Dako), both at 1:500] in 5% NGS in PBST. Sections were probed with primary antibodies for 72 h at 4°C and flipped halfway through the incubation period to allow for maximum penetration of primary antibodies. Secondary antibodies (goat anti-mouse Alexa Fluor 594 (Thermo Fisher Scientific) and goat anti-rabbit Alexa Fluor 647 (Thermo Fisher Scientific), both at 1:1000) were then added to 5% NGS in PBST. Sections were probed with secondary antibodies for 72 h at 4°C. Following incubation with secondary antibodies, sections were washed 3 × 10 min in PBST followed by one wash in 1× PBS. Sections were then mounted onto slides and coverslipped with DAPI Fluoromount-G (Southern Biotech).

### Astrocyte image acquisition and processing

**Figure 1.**
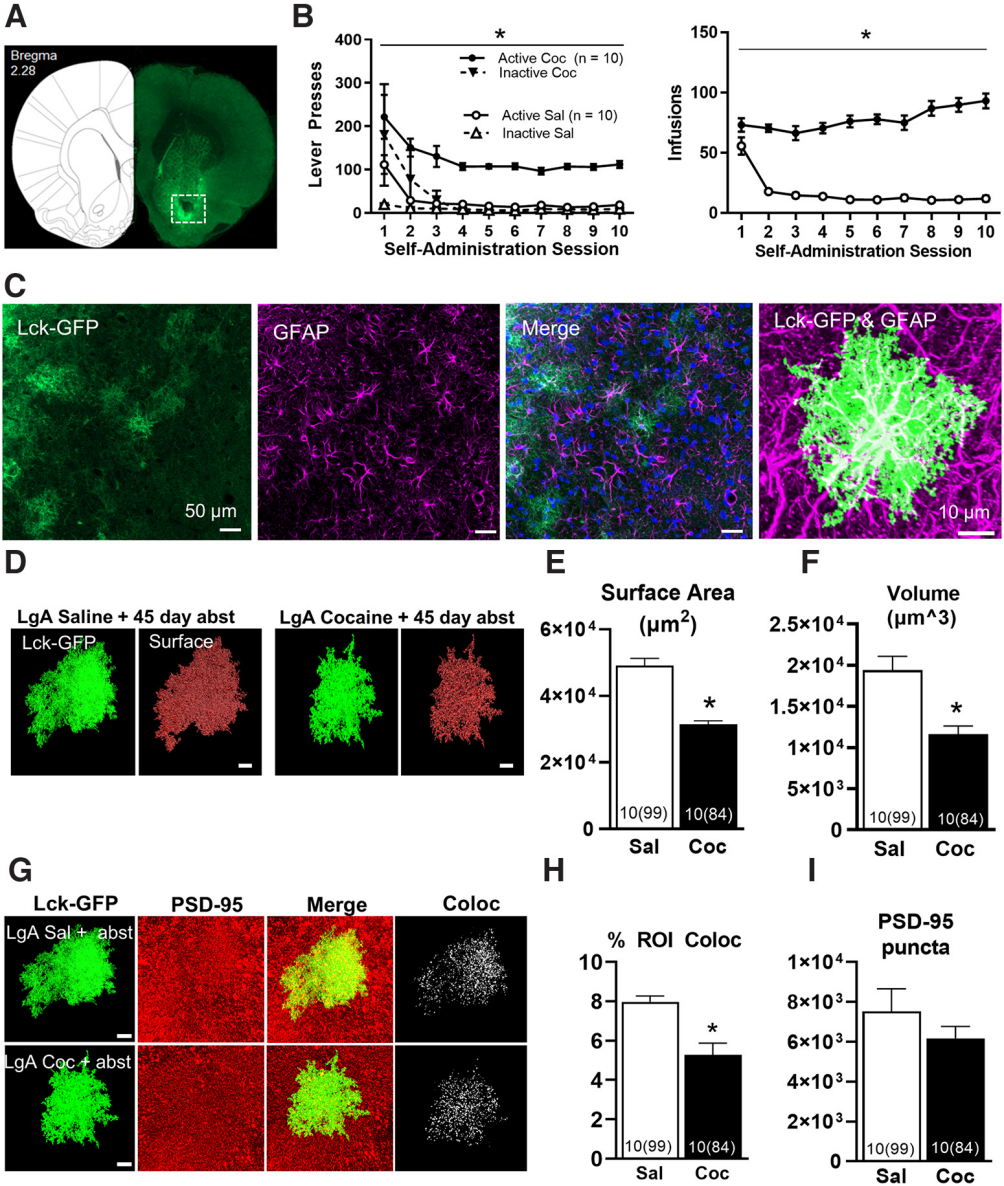
LgA self-administration and 45-d home-cage abstinence results in reduced structural features and synaptic colocalization of NAc astrocytes in male rats. ***A***, Scan of a whole-brain slice containing the NAc, demonstrating expression and spread of AAV5-GfaABC1D-Lck-GFP. Only astrocytes within the white square were selected for further analysis. ***B***, Active lever presses and infusions received during self-administration in male rats. ***C***, Exemplar astrocytes selected for imaging, removed ≥100 μm from the AAV microinjection site. AAV5 GfaABC1D Lck-GFP signal is shown in green (far left), GFAP immunohistochemistry in purple (second panel), DAPI in blue, and merged channels (third panel). A 63× image of an isolated astrocyte selected for analysis with Lck-GFP and GFAP is shown (far right). ***D***, 63× confocal image of a single, isolated Lck-GFP-expressing astrocyte in the NAc (green), with surface reconstruction of isolated astrocytes (red). Surface area (***E***) and volume of astrocytes (***F***) from each group. ***G***, 63× confocal image of an isolated Lck-GFP-expressing astrocyte in the NAc (far left), immunohistochemistry for the synaptic marker PSD-95 (second panel), merge of Lck-GFP and PSD-95 channels (third panel), and colocalization of Lck-GFP-expressing astrocytes with PSD-95 (right), from a saline self-administering rat (top row) and cocaine self-administering rat (bottom row). ***H***, Synaptic colocalization of NAc astrocytes was significantly decreased after LgA cocaine self-administration and prolonged abstinence in male rats, without a statistically significant effect on number of PSD-95-positive puncta (***I***). *p <* *0.05 between groups, all error bars are standard error of the mean (SEM). Scale bar: 10 μm. For all images, *n* represents rats and total cells in parentheses.

Image acquisition and processing of NAc astrocytes were identical to methods described previously (Testen et al., 2018, 2019, 2020). A Zeiss LSM 800 confocal-scanning microscope (405/488/561/640-nm diode lasers, 2 GaAsP detectors, 63× oil-immersed objective), along with the following parameters: 1024 × 1024 pixels, bit depth 16-bit, 4× averaging, 1 μm z-step, was used for image acquisition. Only single, isolated astrocytes within the NAc were acquired (Fig. 1*C*,*D*,*G*). Care was taken to select isolated astrocytes at least 100 μm from the microinjection site, to avoid a possible confound of cellular consequences of AAV microinjection. Astrocytes were not imaged if they were outside of the NAc, bordering other astrocytes, or cut within the z-plane during sectioning.

Following image acquisition, raw images were deconvolved using AutoQuant software (version X3.0.4, MediaCybernetics) and imported into Imaris software (version 8.4.1, Bitplane). Using the Lck-GFP signal from each astrocyte, each cell was reconstructed in 3-dimensions and a surface was built around each astrocyte to obtain measurements of surface area and volume. A masked channel was created to isolate the astrocyte Lck-GFP signal from background and was then used as a region of interest (ROI) to perform colocalization analysis between the masked Lck-GFP signal and PSD-95, represented by the Alexa Fluor 594 signal. A colocalization channel was then generated to obtain the percentage of masked Lck-GFP signal colocalized with PSD-95. For PSD-95 quantification, a fluorescent intensity threshold value was set by taking the average fluorescent intensity value of PSD-95 puncta above the colocalization threshold within the field of view. PSD-95 puncta above this threshold were automatically calculated within a 50 × 50 × 50 μm volume in the center of the image. All image acquisition and analysis were performed blind to groups.

### qPCR for GLT-1 gene expression

Twenty-four hours following the last day of abstinence, female rats were euthanized via rapid decapitation and tissue samples were collected from the NAc. For each animal, 100 μl of RNA from each NAc sample was isolated using the Trizol Plus RNA Purification kit (ThermoFisher). The purity and concentration of RNA was verified on a spectrophotometer. Approximately 100–150 ng of RNA was then used in a reverse transcription assay and converted to cDNA using the High-Capacity cDNA Reverse Transcription kit (Applied Biosciences). For each sample, qPCR amplification was performed in triplicate using the Taqman Fast Advanced Mastermix (Applied Biosciences) in a final volume of 20 μl (3 μl, ∼15 ng of cDNA) under the following conditions: hold for 2 min at 50°C, hold for 2 min at 95°C, and 40 temperature cycles of 1 s at 95°C and 20 s at 60°C. For all samples, GAPDH was used as an endogenous control. All sequences for GLT-1 and GAPDH primers/probes were previously used to assess GLT-1 mRNA levels in the NAc of male rats (R. Kim et al., 2018b): GLT-1A forward: GGAAAGCAACTCTAATCAG/ATG, reverse: CATTGGCCGCCAGAGTTAC, probe: FTCT/AATGCCGCACACAACTCTGTCGQ GLT-1B forward: GGAAAGCAACTCTAATCAG/ATG, reverse: TCCAGGAATGGGAAAGGTAC, probe: FTCT/AATGCCGCACACAACTCTGTCGQ; GAPDH forward: AGGTCGGTGTGA ACGGATTT, reverse: GGCAACAATGTCCACTTTGT, probe: FCGCCTGGTC/TACCAG GGCTGCCQ [F = 5′ Fluorescein (FAM); Q = Quencher (TAMRA)]. Relative concentrations of GLT-1A and GLT-1B were examined using GAPDH as an endogenous control in saline versus cocaine self-administering animals.

### Western blotting for GLT-1 protein expression

Twenty-four hours following the last day of abstinence, female rats were euthanized via rapid decapitation and 1.5-mm punches surrounding NAc tissue was collected. NAc tissue was then homogenized using eight strokes in a hand-driven glass-Teflon homogenizer in 400 μl of sucrose buffer containing 1:100 protease/phosphatase inhibitors (Thermo Fisher Scientific) and 4 μl 0.5 m EDTA (Thermo Fisher Scientific). A P2 membrane subfraction was prepared as previously described (Knackstedt et al., 2010; Reissner et al., 2012) and stored at −80°C until use. On the day of the blotting, P2 pellets were thawed on ice and resuspended in 35 μl of RIPA buffer containing 1% sodium dodecyl sulfate (SDS), protease/phosphatase inhibitors, and EDTA. Samples were centrifuged at 14,000 × *g* for 10 min, and protein concentration for each sample was determined using a BCA assay (Pierce Microplate BCA Protein Assay kit, ThermoFisher). Equal amounts of protein were prepared using 4× NuPage sample buffer (Thermo Fisher Scientific), along with 20 μl of β-mercaptoethanol (Sigma-Aldrich) and heated at 50°C for 30 min before loading on a 10% criterion Tri-HCl gel (Bio-Rad). Samples were then transferred onto PVDF membranes (Millipore Sigma) and membranes were blocked for 1 h at room temperature in Licor Odyssey blocking buffer, then incubated with primary antibodies overnight at 4°C (GLT-1, Millipore AB1783 at 1:4000; Calnexin, Enzo ADI-SPA-860 at 1:4000). Secondary antibody incubation was performed for 1.5 h at 4°C (Licor 800 CW anti-guinea pig at 1:15 000, Licor 680 RP anti-rabbit at 1:15 000). Membranes were then washed 3 × 5 min in Tris-buffered Saline (TBS) + 0.1% Tween and imaged on a Licor Odyssey Fc imager. Optical density for GLT-1 in each sample was divided by optical density for calnexin from the same sample, and expression was normalized to samples from saline-administering rats.

### Data analysis

**Figure 2.**
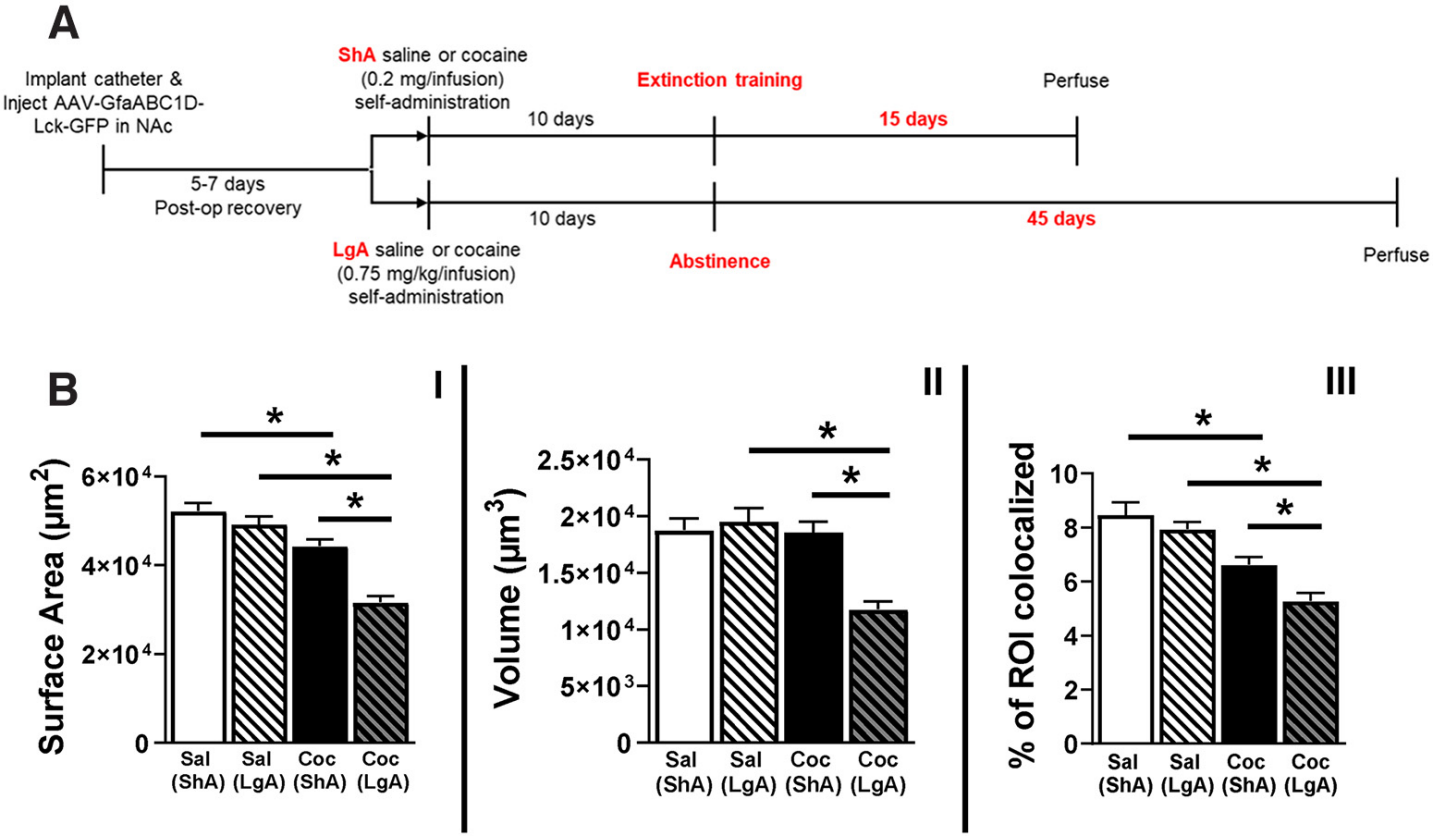
LgA cocaine self-administration followed by 45 d of abstinence results in a greater magnitude of reduced NAc astrocyte structural features and synaptic colocalization in male rats. ***A***, Timeline of behavioral studies for astrocyte analysis. Rats received either 10 d of ShA (2 h/d) or LgA (6 h/d) cocaine or saline self-administration. ShA was followed by 15 d of extinction training, whereas LgA was followed by 45 d of home-cage abstinence. These designs were selected based on their use as two commonly employed rat cocaine self-administration models (Table 1). ***B***, Lck-GFP-labeled NAc core astrocytes were analyzed for surface area (I), volume (II), and colocalization with synaptic marker PSD-95 (III). Note very similar baseline measures observed for astrocytes from saline-administering rats between the two paradigms. However, a significantly greater reduction in all measures was observed after LgA cocaine self-administration followed by prolonged abstinence (right-most bar in all panels). *p < 0.05 as measured by nested ANOVA, error bars are SEM.

All statistical analysis was conducted using SigmaPlot (v.11), SPSS (v. 25), GraphPad Prism (v.8), and SAS (v.9) software. For all self-administration data, a mixed ANOVA (α = 0.05) was performed with drug (saline vs cocaine) and time (self-administration session) set as independent variables. The dependent variable was active lever presses or infusions received during self-administration. For astrocyte imaging data, a nested ANOVA was performed comparing saline versus cocaine self-administering animals, with surface area, volume, synaptic colocalization, or the number of PSD-95 puncta above threshold set as dependent variables. For comparison between cocaine self-administration paradigms (Fig. 2), a nested ANOVA was performed with self-administration paradigm (ShA/extinction vs LgA/abstinence) set as a factor. For qRT-PCR, the ΔΔCt method was used for relative comparisons of GLT-1A and GLT-1B (with GAPDH used as an endogenous control) in cocaine versus saline self-administering animals (H.S. Kim et al., 2002; R. Kim et al., 2018b). For GLT-1 protein levels, relative GLT-1 levels were determined using a ratio of GLT-1 to calnexin. These values were then normalized to saline self-administering animals to determine relative change in GLT-1 protein expression in cocaine self-administering rats. For both GLT-1 protein and mRNA experiments, a two-tailed unpaired *t* test was conducted to examine differences between saline and cocaine self-administering animals.

**Table 1:** Experiments using the ShA/extinction and the LgA/abstinence models of rodent cocaine self-administration

Protocol	Behavioral design	References
ShA/extinction	FR1 schedule, 2 h/d 10–12 d of self-administration 10–20 d of extinction	Baker et al. (2003), Knackstedt et al. (2010), Moussawi et al. (2011), LaLumiere et al. (2012), Trantham-Davidson et al. (2012), Cosme et al. (2015, 2018), Reissner et al. (2015), Scofield et al. (2016), LaCrosse et al. (2017), Bystrowska et al. (2018), Kim et al. (2018b), Logan et al. (2018), Sepulveda-Orengo et al. (2018), Testen et al. (2018), Smaga et al. (2020)
LgA/abstinence	FR1 schedule, 6 h/d 10 d of self-administration 30+ d of abstinence	Lu et al. (2004a, 2005a,b), Conrad et al. (2008), Koya et al. (2009), Fischer-Smith et al. (2012), Karlsson et al. (2013), Loweth et al. (2014), Christian et al. (2017), Kim et al. (2018b), Stefanik et al. (2018), Corbett et al. (2021), Alonso et al. (2022)

## Results

### Morphology and synaptic co-localization of NAc astrocytes is significantly decreased in male rats following LgA cocaine self-administration and prolonged abstinence

Figure 1*A* indicates the region of AAV5-GfaABC1D-LckGFP microinjection; cells for analysis were selected within the NAc core. Active lever presses and infusions are shown in Figure 1*B*. Cocaine self-administering rats demonstrated a significantly greater amount of both active lever presses (*F*_(1,199)_ = 172, *p *<* *0.001; Fig. 1*B*, left) and number of infusions received (*F*_(1,199)_ = 205.33, *p *< 0.001; Fig. 1*B*, right). Figure 1*C* shows representative astrocytes at 20× (first three panels) and 63× (fourth, right panel) taken for analysis, beyond 100 μm or more from the site of microinjection. Note the classic bushy shape of astrocytes, and absence of evidence of reactive gliosis. To verify that analysis was specific to astrocytes, all slices were probed for GFAP, an astrocyte-specific marker as well as for the nuclei marker DAPI. Compared with NAc core astrocytes from saline self-administering rats, astrocytes from rats following 45 d of abstinence from LgA cocaine self-administration showed a significant decrease in both surface area (*F*_(1,18)_ = 58.67, *p *<* *0.001; Fig. 1*D*,*E*) and volume (*F*_(1,18)_ = 30.26, *p *<* *0.001; Fig. 1*D*,*F*).

To assess synaptic co-localization of NAc astrocytes, co-localization between the postsynaptic marker PSD-95 and the masked Lck-GFP channel was used to generate a new co-localization channel (Fig. 1*G*). After 45 d of abstinence following LgA cocaine self-administration, the co-localization between Lck-GFP and PSD-95-positive voxels were significantly lower than co-localization between the two channels in animals that had previously self-administered saline (*F*_(1,18)_ = 48.04, *p *<* *0.001; Fig. 1*G*,*H*). One possible explanation for the decrease in synaptic colocalization is an effect of LgA cocaine self-administration and prolonged abstinence on NAc PSD-95. Although PSD-95-positive pixels from rats that underwent prolonged abstinence from LgA cocaine self-administration exhibit a modest decrease that approaches statistical significance, this decrease was not statistically significant (*F*_(1,18)_ = 4.37, *p *=* *0.051; Fig. 1*I*).

Since the values for measurements from LgA saline-administering rats were similar and not significantly different from those previously observed following ShA (2 h/d) and extinction training (Testen et al., 2018), we compared measurements from the current study (LgA followed by 45 d of abstinence) with those previously reported (Testen et al., 2018). Table 1 provides a comparison of the variables used in these two different behavioral models, as well as representative citations in which these models have been previously employed. Figure 2*A* provides a comparison of the behavioral models used before tissue preparation for astrocyte analysis. Similarly to the previously reported comparative changes in downregulation of NAc GLT-1 expression following limited versus extended cocaine self-administration access and abstinence (Fischer-Smith et al., 2012; R. Kim et al., 2018b), the changes in NAc astrocytes were more pronounced following LgA cocaine self-administration and prolonged abstinence. Compared with rats that received ShA cocaine/extinction, NAc astrocytes from rats that received LgA cocaine/abstinence exhibit a smaller surface area (*F*_(1,17)_ = 37.17, *p *=* *0.0001; Fig. 2*B*, I) and volume (*F*_(1,17)_ = 35.26, *p *=* *0.0001; Fig. 2*B*, II), as well as reduced synaptic colocalization (*F*_(1,17)_ = 11.38, *p *=* *0.0036; Fig. 2*B*, III).

**Figure 3.**
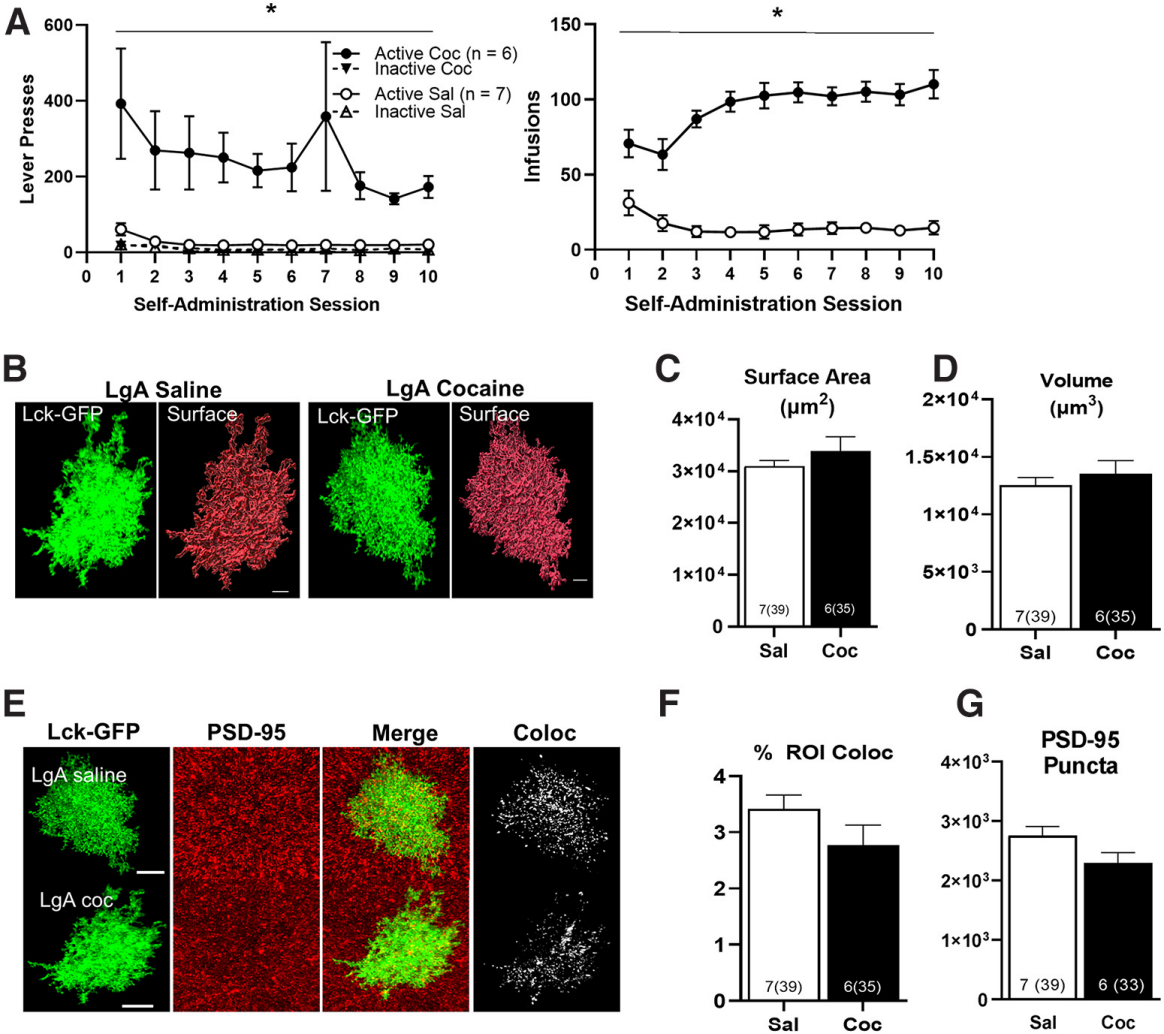
LgA cocaine self-administration has no effect on NAc astrocytes on withdrawal day 1 (WD 1) in male rats. ***A***, Active lever presses and infusions received during self-administration. ***B***, Representative 63× image of a Lck-GFP labeled NAc astrocyte and surface rendering from a saline (left panels) and cocaine self-administering rat (right panels). ***C***, There was no significant difference between saline and cocaine self-administering rats in surface area and (***D***) volume of NAc astrocytes on WD 1. ***E***, Lck-GFP labeled astrocytes (far left), immunohistochemistry for PSD-95 (second panels), merged image between the two channels (third panels) and colocalized puncta (far right panels) in a saline (top row) and cocaine (bottom row) self-administering rat. ***F***, There was no significant difference between saline and cocaine self-administering rats in synaptic colocalization of NAc astrocytes on WD 1 or (***G***) total number of PSD-95 puncta above threshold. *p < 0.05 between groups, error bars are SEM. For all images, *n* represents rats and total cells in parentheses.

Given the notable magnitude of reductions in structural features and synaptic colocalization observed at withdrawal day (WD), it was of interest to determine whether these effects emerged as a function of self-administration or developed across abstinence. We have previously reported that following 10 d of ShA (2 h/d) self-administration, reductions are observed following 15 d of extinction, but not 24 h after the last self-administration session (Testen et al., 2018). To examine the effects of abstinence in the LgA model, a separate cohort of rats received intra-NAc AAV5-GfaABC1D-Lck-GFP and was trained in self-administration identically as described for those in Figure 1. Twenty-four hours following 10 d of LgA self-administration (Fig. 3*A*), tissue was extracted for analysis. No significant differences were observed in surface area (*F*_(1,11)_ = 1.04, *p *=* *0.329; Fig. 3*B*,*C*), volume (*F*_(1,11)_ = 0.21, *p *=* *0.653; Fig. 3*B*,*D*) or synaptic colocalization with PSD-95 (*F*_(1,11)_ = 3.39, *p *=* *0.093; Fig. 3*E*,*F*).

### Morphology and synaptic co-localization of NAc astrocytes is unaffected in female rats following LgA cocaine self-administration and prolonged abstinence

**Figure 4.**
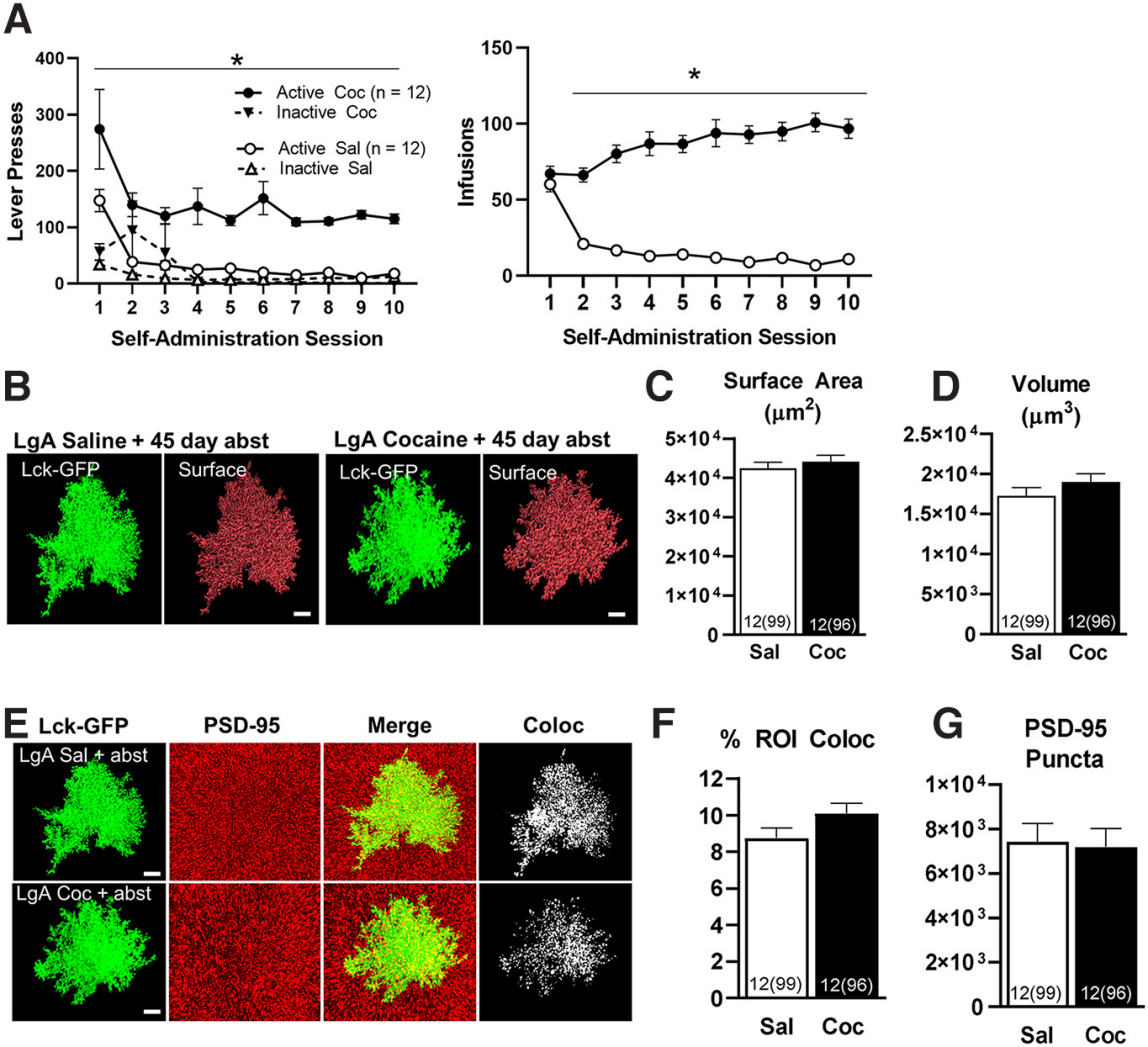
Cocaine self-administration and abstinence has no effect on surface area, volume, or synaptic colocalization of NAc astrocytes in female rats. ***A***, Active lever presses and infusions received during self-administration. ***B***, 63× confocal image of a single, representative isolated Lck-GFP-expressing astrocyte in the NAc (green), along with surface reconstruction (red) from each group. ***C***, No differences were observed in surface area or (***D***) volume of astrocytes. ***E***, 63× confocal image of an isolated Lck-GFP-expressing astrocyte in the NAc (far left), immunohistochemistry for the synaptic marker PSD-95 (second panel), merge of Lck-GFP and PSD-95 channels (third panel), and colocalization of Lck-GFP-expressing astrocytes with PSD-95 (far right), from a saline self-administering rat (top row) and cocaine self-administering rat (bottom row). Synaptic colocalization (***F***) and the number of PSD-95-positive puncta (***G***) above threshold were not different between saline and cocaine self-administering female rats. *p < 0.05 between groups, error bars are SEM. For all images, *n* represents rats and total cells in parentheses.

Behavioral data for all female rats used in the NAc astrocyte imaging experiment are shown in Figure 4*A*. Cocaine self-administering female rats showed a significantly greater number of both active lever presses (*F*_(1,239)_ = 54.45, *p *<* *0.001; Fig. 4*A*, left) and number of infusions received (*F*_(1,239)_ = 190.59, *p *<* *0.001; Fig. 4*A*, right) in comparison to saline self-administering female rats.

Single-cell analysis of NAc astrocytes in female rats was performed identically as for male rats. Although astrocytes from the NAc of male rats exhibited a pronounced decrease in both surface area (Fig. 1*D*) and volume (Fig. 1*E*) at WD 45, this decrease was not observed in NAc astrocytes from female rats. No significant differences were observed in surface area (*F*_(1,22)_ = 0.72, *p *=* *0.40; Fig. 4*B*,*C*) or volume (*F*_(1,22)_ = 1.51, *p *=* *0.23; Fig. 4*B*,*D*) between saline and cocaine self-administering female rats following 45 d of abstinence. Furthermore, while male rats also exhibited a significant decrease in synaptic co-localization of NAc astrocytes, this decrease was not observed in female rats (*F*_(1,22)_ = 4.16, *p *=* *0.054; Fig. 4*E*,*F*). There was also no effect of cocaine on the number of positive PSD-95 puncta above threshold in female rats (*F*_(1,22)_ = 0.08, *p *=* *0.77; Fig. 4*E*,*G*). To examine a possible role for the estrous cycle in modulating these results, saline and cocaine self-administering female rats were further divided into groups based on estrous cycle phase. While stages of the estrous cycle were not evenly distributed between rats, there was no indication of possible effect of estrous cycle stage on surface area or volume of NAc astrocytes (data not shown). Furthermore, there was no effect of estrous cycle stage on synaptic co-localization with PSD-95 or the number of positive PSD-95 puncta above threshold (data not shown).

### GLT-1 mRNA and protein levels are unchanged in the NAc of female rats following LgA cocaine self-administration and prolonged abstinence

**Figure 5.**
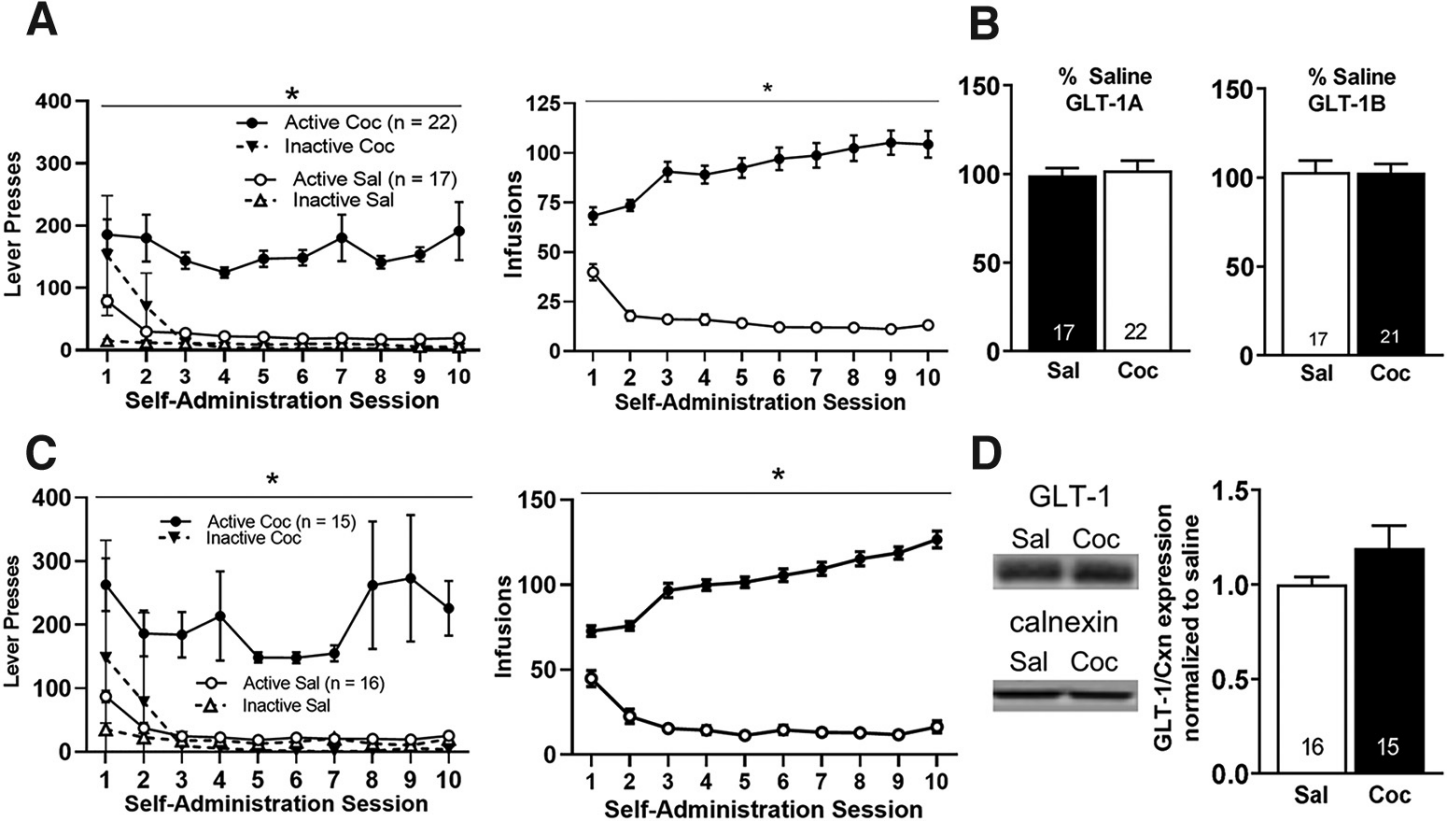
GLT-1 expression is unchanged in the NAc of female rats following LgA cocaine self-administration and 45 d home-cage abstinence. ***A***, Active lever presses and infusions received during self-administration sessions in saline versus cocaine self-administering female rats used to assess changes in NAc GLT-1 gene expression. ***B***, No significant differences were observed in NAc GLT-1A or GLT-1B mRNA levels in female rats following LgA cocaine self-administration and 45 d of abstinence. ***C***, Active lever presses and infusions received during self-administration sessions in saline versus cocaine self-administering female rats used to assess changes in NAc GLT-1 protein expression. ***D***, No significant changes were observed in NAc GLT-1 protein levels in female rats following LgA cocaine self-administration and 45 d abstinence. *p < 0.05 between groups, error bars are SEM.

Previous studies by our lab and others have demonstrated a marked decrease in both GLT-1 mRNA and protein levels in the NAc of male rats following LgA cocaine self-administration and prolonged abstinence (Fischer-Smith et al., 2012; R. Kim et al., 2018b). While a decrease in protein expression of GLT-1 has been reported in female rat NAc following ShA self-administration and extinction, neither mRNA nor protein levels have been explored in females subsequent to LgA self-administration. Accordingly, we performed these measurements in female rats, as described above for males. Behavioral data for all female rats used to assess NAc GLT-1 mRNA and protein levels is shown in Figure 5*A*,*C*. Compared with saline self-administering female rats, cocaine-administering female rats for GLT-1 mRNA analysis exhibited a significant increase in both active lever presses (*F*_(1,389)_ = 52.42, *p *<* *0.001; Fig. 5*A*, left) and number of infusions received (*F*_(1,389)_ = 172.56, *p *<* *0.001; Fig. 5*A*, right). No significant differences between groups in NAc mRNA levels of the dominant splice isoform of GLT-1, GLT-1A were observed at WD 45 (*t*_(37)_ = 0.42, *p *=* *0.68; Fig. 5*B*, left) or splice variant GLT-1B (*t*_(36)_ = 0.04, *p *= 0.97; Fig. 5*B*, right). Similarly, as for astrocyte structural analysis, subdividing female rats on the basis of estrous cycle stage did not reveal an effect (data not shown).

Compared with saline self-administering female rats, cocaine self-administering female rats for Western blot analysis exhibited a significant increase in both active lever presses (*F*_(1,309)_ = 43.34, *p *<* *0.001; Fig. 5*C*, left) and number of infusions received (*F*_(1,309)_ = 616.08, *p *<* *0.001; Fig. 5*C*, right). Following 45 d of abstinence from LgA saline or cocaine self-administration, there was no significant difference between groups in NAc GLT-1 protein levels (*t*_(29)_ = −1.62, *p *=* *0.12; Fig. 5*D*). Furthermore, when saline and cocaine self-administering female rats were further divided into groups based on estrous cycle stage, there was also no effect of estrous cycle stage on NAc GLT-1 protein expression (data not shown).

## Discussion

Similarly to previous findings after ShA cocaine self-administration and extinction training (Scofield et al., 2016; Testen et al., 2018), results from the current study indicate that following 10 d of LgA cocaine self-administration and 45 d of prolonged abstinence, NAc astrocytes exhibit a marked decrease in surface area, volume, and synaptic colocalization. Notably, these reductions are significantly greater than those previously observed following ShA and extinction (Testen et al., 2018). In contrast, no effect of LgA cocaine was observed 24 h after the last self-administration session in male rats. Furthermore, no effect of cocaine was observed on NAc astrocyte morphology, or synaptic colocalization of NAc astrocytes, NAc GLT-1 mRNA, or protein expression in female rats. Accordingly, the effects of cocaine on NAc astrocytes are specific to males, and are exacerbated across abstinence.

### Astrocytes from the NAc of male rats exhibit a pronounced retracted phenotype following prolonged abstinence from LgA cocaine self-administration

Previous reports from our lab and others have revealed that astrocytes from the NAc of male rats exhibit reductions in structural features and synaptic colocalization following ShA self-administration and extinction from cocaine, methamphetamine, and heroin (Scofield et al., 2016; Testen et al., 2018; Kruyer et al., 2019; Siemsen et al., 2019). Results from the present study indicate pronounced (∼40%), abstinence-dependent reductions in the morphometric properties and synaptic colocalization of NAc astrocytes following prolonged abstinence from LgA cocaine self-administration, as no effect of cocaine was observed 24 h following the last self-administration session on WD 1. This result extends findings from previous studies which have demonstrated progressive adaptations subsequent to LgA cocaine self-administration across abstinence. For example, maturation of silent synapses and surface expression of calcium permeable AMPA receptors occur progressively across abstinence subsequent to LgA self-administration (Conrad et al., 2008; Wolf, 2016; Dong et al., 2017). Similarly, decreases in expression of NAc GLT-1 protein and gene expression occur as a function of both duration of self-administration, as well as duration of abstinence (Fischer-Smith et al., 2012; R. Kim et al., 2018b).

When making a direct comparison between measures of NAc astrocytes taken following ShA self-administration and extinction, versus LgA self-administration and home-cage abstinence, we find a greater magnitude of decrease in the surface area, volume and synaptic colocalization of NAc astrocytes associated with the latter (Fig. 2). It is important to note, however, that several variables exist between these self-administration models, and accordingly we cannot determine whether the exacerbated effects observed following prolonged home-cage abstinence are a function of cocaine access, extinction versus abstinence, or duration of withdrawal. Despite the existing variables between these models, we selected these because they are commonly employed in rat self-administration studies in the literature (Table 1). Moreover, we have previously found that while LgA cocaine self-administration followed by prolonged abstinence results in decreased GLT-1 gene expression, ShA is without effect when followed by either extinction or home-cage abstinence for either 14 or 45 d (R. Kim et al., 2018b). Accordingly, it is most likely that an interaction between duration of cocaine access together with duration of abstinence is responsible for the exacerbated effects observed in the present study. Relatedly, both duration of self-administration and abstinence have been previously reported to contribute to progressive effects on NAc GLT-1 protein expression (Fischer-Smith et al., 2012). These findings collectively indicate the likelihood that drug access and abstinence periods lead to an exacerbated effect of LgA self-administration and prolonged abstinence on structural features of NAc astrocytes.

### Astrocytes from the NAc of female rats exhibit no change in surface area, volume, synaptic colocalization, or GLT-1 expression following abstinence from LgA cocaine self-administration

Despite reductions in structural features and synaptic colocalization of astrocytes, and expression of GLT-1 in male rats (Knackstedt et al., 2010; Fischer-Smith et al., 2012; R. Kim et al., 2018b), no effects of cocaine self-administration and abstinence were observed on any of these measures in the NAc of female rats. We did not directly assess the effect of cocaine self-administration on GLT-1 expression in male rats in the current study, as this has been demonstrated previously in a number of reports across several laboratories (Knackstedt et al., 2010; Fischer-Smith et al., 2012; Fischer et al., 2013; Reissner et al., 2014, 2015; LaCrosse et al., 2017; R. Kim et al., 2018b; Sepulveda-Orengo et al., 2018) Most specifically, three of the citations above analyze GLT-1 expression (mRNA or protein) at a 45 d abstinence time point following LgA self-administration in male rats (Fischer-Smith et al., 2012; Fischer et al., 2013; R. Kim et al., 2018b). This sex difference suggests a possible protective role of estrogens and other sex hormones against the cocaine-induced changes in NAc astrocytes, as well as against the cocaine-induced downregulation in GLT-1. In contrast, however, a decrease in NAc GLT-1 protein expression has been reported in female rats following cocaine ShA self-administration and extinction, independent of estrous stage (Bechard et al., 2018). However, an effect of estrous cycle stage was observed on the efficacy of ceftriaxone to inhibit reinstatement behavior, suggesting sex and estrous stage-dependent effects of GLT-1 modulators on cocaine seeking.

Reports in the literature collectively indicate a complex relationship between sex and astrocyte-mediated glutamate transporter expression and activity. Numerous studies *in vitro* have shown that estrogens can positively regulate expression of glutamate transporters including GLT-1 (Liang et al., 2002; Pawlak et al., 2005). For example, estradiol and tamoxifen act as neuroprotective agents by increasing expression of glutamate transporters and normalizing glutamate uptake in pathologic conditions (Shi et al., 1997; Kimelberg et al., 2000; Liang et al., 2002; Pawlak et al., 2005; Lee et al., 2009). Studies in rat spinal cord indicate that EAAT activity is lower in females than males, particularly during the estrus stage, independent of GLT-1 expression (Sajjad et al., 2016). Since GLT-1 is primarily expressed on astrocytes, it remains possible that sex hormones can also protect against the cocaine-induced changes in the morphology and synaptic colocalization of NAc astrocytes. However, very few studies have been conducted to assess the effect of sex on astrocytes. Interestingly, a recent report on sex similarities and differences in astrocytes in response to chronic stress found that chronic unpredictable stress resulted in decreased GFAP expression in the PFC of male mice (akin to numerous other chronic stress models; for review, see R. Kim et al., 2018a), without effect in females (Woodburn et al., 2021). Moreover, a single nucleus RNA sequencing experiment demonstrated cocaine induced changes in NAc gene expression in astrocytes, which differed based on sex (Savell et al., 2020). This result adds to a growing literature of sex-dependent changes in astrocytes. How these changes are related to the observed sex-dependent effects of cocaine (discussed below) remains an area for future research.

### Sex-dependent effects of cocaine in the brain

The current study adds to the growing list of sexually divergent effects of drugs of abuse on the brain (for review, see Becker and Chartoff, 2019; Giacometti and Barker, 2020; Kokane and Perrotti, 2020). Notably, sex differences and estrogen-dependent effects have been observed among the cellular consequence of cocaine in the NAc, including magnitude of dopamine release (Calipari et al., 2017), and changes in protein (López et al., 2021) and gene expression (Savell et al., 2020).

Accordingly, although female rats display both incubation of cocaine craving and reinstatement behavior as do male rats, distinct mechanisms may be driving similar behaviors (Zlebnik and Carroll, 2015; Nicolas et al., 2019, 2022; Fredriksson et al., 2021). For example, several studies have indicated effects of estrous stage on measures which can influence cocaine seeking, including surface GluA1 AMPA receptor expression (Bechard et al., 2018). Furthermore, the spine density of NAc medium spiny neurons (MSNs) and miniature EPSC frequency of NAc MSNs is elevated in female versus male rats in response to intraperitoneal injections of cocaine (Wissman et al., 2011). Other studies have shown increased NAc protein levels of CREB and PKA following cocaine administration in male versus female rats (Nazarian et al., 2009). However, how these systems interact with the estrous cycle to produce neurobiological adaptations that drive cocaine-seeking in female rats remains to be studied. Possible avenues to address this directly could include administration of estradiol or estrogen receptor agonists into the NAc of male rats for assessment of protection for astrocyte morphology and synaptic colocalization, or use of ovariectomized females for cocaine self-administration and assessment of effects on astrocytes.

### Functional consequences of impaired astrocyte structure

Several lines of evidence indicate that NAc astrocytes may exert an inhibitory influence over drug seeking. For example, Gq DREADD agonism of NAc astrocytes can inhibit cocaine reinstatement (Scofield et al., 2015), as well as ethanol self-administration (Bull et al., 2014). Moreover, Corkrum and colleagues have shown that agonism of NAc astrocyte via Gq DREADDs in acute brain slices results in depression of EPSCs in MSNs (Corkrum et al., 2020). Activation of Gq signaling in astrocytes leads to release of ATP hydrolyzed to adenosine, which inhibits glutamatergic transmission (Durkee et al., 2019; Kofuji and Araque, 2021). Accordingly, we propose that impaired structure and function of NAc astrocytes results in disinhibition of NAc synapses, contributing to the excitatory synaptic drive associated with relapse propensity and drug seeking. However, it is currently unknown whether these observations would also extend to female rats, or exhibit sex dependence. These studies are necessary to determine whether the inhibitory effect of NAc astrocytes on drug seeking behaviors is conserved or variable between sexes, contributing to the body of literature indicating sexually divergent mechanisms of drug relapse vulnerability.

### Conclusions and future directions

The results from the present studies provide insight into the effects of prolonged abstinence from LgA cocaine self-administration on NAc astrocytes. Results show that in male rats, NAc astrocytes exist in a significantly retracted state at WD 45, but not at WD 1. The findings from this study also indicate that in female rats, abstinence from LgA cocaine self-administration had no effect on astrocytes and further, had no effect on NAc GLT-1 expression. Moreover, there was no evidence of correlation between estrous cycle stage and cocaine-induced changes in NAc GLT-1 expression or NAc astrocytes, although not all stages were sufficiently powered for a rigorous analysis. Interestingly, direct comparison of two commonly employed rat self-administration models (Table 1) indicates significantly greater effects of the LgA/abstinence model as compared with the ShA/extinction model. However, as previously noted, these are two separate models, which differ across a number of variables. Notably, both models use an FR1 schedule of reinforcement. Future studies will also inform whether other schedules of reinforcement, including intermittent access, may have similar or different effects. Future experiments will also be critical for understanding the relationship between sex and glial cell function. The signaling pathways and mechanisms that contribute to this phenomenon remain to be discovered and represent important areas for future research.

## Synthesis

Reviewing Editor: Leandro Vendruscolo, The Scripps Research Institute Decisions are customarily a result of the Reviewing Editor and the peer reviewers coming together and discussing their recommendations until a consensus is reached. When revisions are invited, a fact-based synthesis statement explaining their decision and outlining what is needed to prepare a revision will be listed below. The following reviewer(s) agreed to reveal their identity: NONE. Note: If this manuscript was transferred from JNeurosci and a decision was made to accept the manuscript without peer review, a brief statement to this effect will instead be what is listed below.

N/A
